# Renal pathological changes after successful treatment of LCDD using cyclophosphamide, thalidomide, and dexamethasone

**DOI:** 10.1080/0886022X.2021.1988967

**Published:** 2021-10-17

**Authors:** Di Wang, Yan Wang, Shiren Sun

**Affiliations:** Department of Nephrology, Xijing Hospital, the First Affiliated Hospital of Fourth Military Medical University, Xi’an, China

Dear Editor,

Until now, there is no evidence-based consensus on the treatment for light chain deposition disease (LCDD). Some anti-myeloma agents, such as bortezomib-based, melphalan-based, or thalidomide/lenalidomide-based regimens, are concerned [1–4]. The low-cost regimen of cyclophosphamide, thalidomide, and dexamethasone (CTD) has not been reported in treatment for LCDD. Here we report an LCDD patient with nodular sclerosis on renal biopsy, then successfully treated with CTD regimen, and the second kidney biopsy revealed that the nodular sclerosis was significantly reduced with a complete hematological response (CR).

A 51-year-old woman presented with lower extremities and eyelids edema for 6 months and abdominal distension for 1 month. She had a history of hypertension for 6 years, the highest blood pressure was 200/106 mmHg. There was no family history of kidney disease and diabetes mellitus. On admission, 24 h-proteinuria was 2840 mg, serum albumin was 26.7 g/L, blood urea nitrogen was 13 mmol/L, serum creatinine was 125 μmol/L, eGFR was 44.7 mL/min/1.73 m^2^; serum free light chain: *κ* was 54.09 mg/L, *λ* was 47.49 mg/L, *κ*/*λ* was 1.14; serum immunofixation electrophoresis: IgG lambda monoclonal band. Bone marrow aspiration examination showed that the marrow was normocellular with 2% atypical plasma cells. Antinuclear antibodies, anti-phospholipase A2 receptor (PLA2R) antibodies were negative.

**Figure 1.**
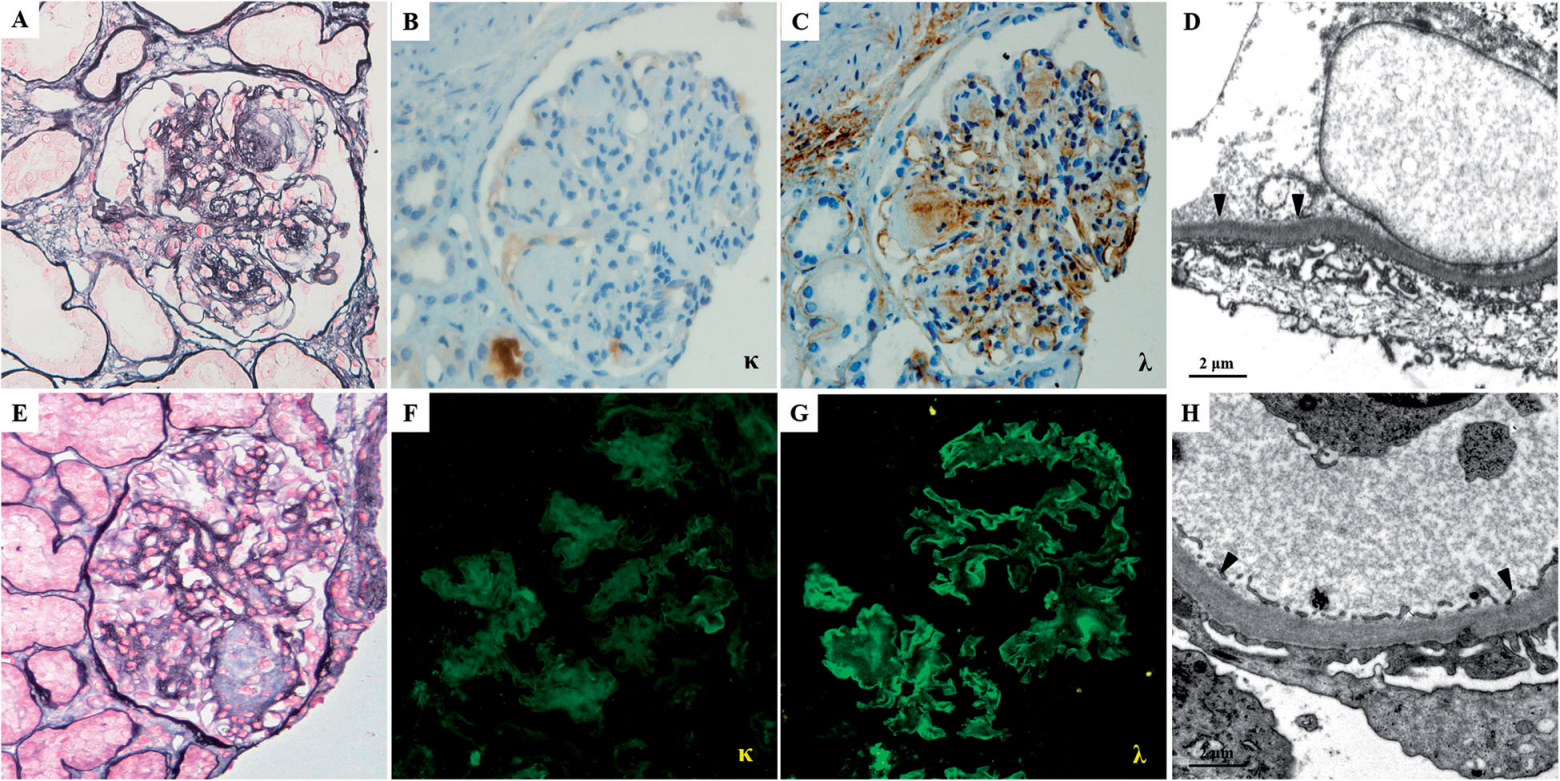
The pathological findings of the first (A–D) and second (E–H) renal biopsy. (A) The glomeruli exhibit sclerotic mesangial nodules with severe mesangial hypercellularity (PASM, ×400). (B) Kappa light chain is negative by immunohistochemistry staining on paraffin tissue (×400). (C) Lambda light chain deposit in nodular sclerosis area and along capillary wall and TBM by immunohistochemistry staining on paraffin tissue (×400). (D) Electron microscopy of formalin-fixed renal tissue. The granular dense (arrowhead) deposits along the inner side of GBM (×12,000). (E) The nodular sclerosis was significantly reduced than the first biopsy (PASM, ×400). (F) The kappa-light chain stains negatively. (G) Immunofluorescence staining showed that the lambda-light chain is positive along the capillary wall of glomeruli (×400). (H) Electron microscopy. There is trace granular (arrowhead) dense deposition along the inner side of GBM (×12,000).

The patient underwent the first renal biopsy. Light microscopy (LM) showed severe mesangial expansion with the profound mesangial nodular formation in 18/20 glomeruli, and the glomerular basement membrane (GBM) was irregularly thickened with a double contour. Congo red staining was negative. Renal tubules presented minute atrophy, and the renal interstitium was mildly fibrotic with a small focus of monocyte, which extended less than 5% of the cortical area. Sclerotic change of small arteries was mild. Being absent of glomeruli in frozen tissue and glutaraldehyde fixed tissue, the formalin-fixed renal tissue was stained for immunohistochemical and re-fixed for electron microscopic examination. Immunohistochemical staining revealed a single lambda light chain deposit in the mesangial region and linearly along the GBM, tubular basement membrane (TBM), and Bowman’s capsules. There was no staining for the kappa light chain, IgG, IgA, and IgM. Electron microscopy (EM) showed that the massive granular and amorphous electron-dense deposits along the inner side of GBM, and the outer side of TBM. Final pathological diagnosis: lambda-type LCDD (details in Figure 1(A–D)).

The patient was treated with CTD regimens (28d per cycle) as follows: dexamethasone (20 mg orally days 1–4 days, 15–18 days) with cyclophosphamide (500 mg IV on days 1, 8, and 15) and oral thalidomide 50 mg daily (increased to 200 mg if well-tolerated). The CR and renal complete remission were reached after 14 cycles of chemotherapy, serum-free light chain: *κ* was 10.60 mg/L, *λ* was 15.40 mg/L, *κ*/*λ* was 0.68. The 24-h proteinuria reduced to 130 mg and the plasma albumin rose to 47.3 g/L, and the eGFR was stable. The monoclonal lambda light chain protein became undetectable in her serum and urine by immunofixation. Then, the patient was followed by thalidomide 200 mg per day for maintenance treatment.

At the 29-month follow-up, this patient underwent a repeat renal biopsy. The biopsy specimen contained 19 glomeruli, 5 were ischemic sclerosis. The rest non-sclerotic 14 glomeruli indicated the mesangial matrix was significantly reduced than the first biopsy, 11 of which revealed a complete resolution of the nodular lesions. The tubular atrophy and interstitial fibrosis occupied approximately 25% of the cortical region, around glomeruli with ischemic sclerosis, and moderate hyaline degeneration was found in arterioles, which was slightly exacerbated than the first biopsy in the pathological change of tubulointerstitial and vascular. That was maybe due to the poor controlling of blood pressure. EM also revealed a marked reduction of the electron-dense deposits, only trace granular deposits along the inner side of GBM and the outer side of TBM. IF showed staining for the lambda light chain was still detected along the GBM and TBM.

LCDD is a rare Monoclonal gammopathy of renal significance (MGRS), which often results in renal insufficiency associated with either nephrotic syndrome or asymptomatic proteinuria. The pathological features are nodular glomerulosclerosis and granular electron-dense deposits on the GBM and TBM. To date, there is no guideline for the treatment of LCDD. The therapeutic approach is similar to AL amyloidosis, controlling the underlying plasma cell clone using chemotherapy or autologous hematopoietic cell transplantation. Deep hematologic response, achieved early in the disease course, predicts favorable renal and overall outcomes. In a nationwide cohort study, 169 patients were treated with chemotherapy agents, including bortezomib (58%), alkylators (17%), thalidomide or lenalidomide (10%), adriamycin (9.5%), rituximab (2.4%), or steroids alone (<1%). The overall response rate was 67% and CR was 30%. 3-year renal survival was 86 versus 62 percent in patients with and without VGPR/CR respectively, and 91 versus 63 percent in renal responders versus non-responders [5]. But until now, there is no report about CTD in the treatment of LCDD, our study indicated that CTD chemotherapy can reach a deep hematologic response and following with complete renal response.

Serial-free light chain assays and/or immunofixation electrophoresis can be used to monitor hematologic response to chemotherapy. How the involved tissue load of light chain deposition changes is unclear. Some case reports showed that treatment with melphalan and prednisolone (MP) therapy led to a hematological response, serial evaluations of renal histology revealed the resolution of nodular lesions [6]. However, MP therapy is one of the classical chemotherapy regimens for multiple myeloma, and intensive chemotherapy was more toxic in patients with LCDD compared with those with multiple myeloma [7]. Another report showed that treatment with high-dose melphalan and autologous stem cell transplantation (ASCT), led to control of monoclonal free light chain production, and the nodular lesions seen in the first biopsy were not present in the new one [8], but high-dose chemotherapy often can be accompanied by severe toxicity. In our study, the patient underwent the repeated biopsy after CR and renal remission, renal pathological features also indicate that the almost disappearance of multi-nodular sclerosis in LM and the reduction of dense deposits by EM. Though light chains on IF persistent existence. This case indicates that the deep hematologic response can reduce the deposition and toxicity of the pathogenic light chain, and the nodular lesions can be reversed to the initial stage. But the underlying mechanism is unknown and needs to be investigated further.

This case highlights the need to target at least a hematologic VGPR (very good partial response) with chemotherapy, even among patients with renal dysfunction and advanced renal pathological injury, to reduce the proteinuria and delay progression to end-stage renal disease (ESRD). And the cost-effective regimen, CTD, may be a good choice.
